# The expression of *CBF* genes at *Fr-2* locus is associated with the level of frost tolerance in Bulgarian winter wheat cultivars

**DOI:** 10.1080/13102818.2014.944401

**Published:** 2014-09-25

**Authors:** Elena Georgieva Todorovska, Stanislav Kolev, Nikolai Kirilov Christov, Andras Balint, Gabor Kocsy, Attila Vágújfalvi, Gabor Galiba

**Affiliations:** ^a^ AgroBioInstitute, Agricultural Academy, Sofia, Bulgaria; ^b^ Agricultural Institute, Centre for Agricultural Research, Hungarian Academy of Sciences, Martonvásár, Hungary

**Keywords:** wheat (*T. aestivum* L.), frost tolerance, cold acclimation, *CBF* (*C-repeat binding factors*) genes, gene expression

## Abstract

The regulation of the majority of cold-regulated genes in plants is mediated by *CBF* (*C-repeat binding factors*) transcription factor family. Natural differences in frost tolerance (FT) of wheat have been mapped to the *Fr-2* (*Frost Resistance-2*) locus on chromosome group 5 and are associated with variation in threshold induction temperatures and/or transcript levels of *CBF* genes.

This study used real-time reverse-transcription polymerase chain reaction (qRT-PCR) to compare the relative expression levels of four *T. aestivum CBF* genes (*TaCBF15.2, TaCBFA19, TaCBFA2* and *TaCBFD21*) in crown tissue of two Bulgarian hexaploid winter wheat cultivars (Milena and Russalka) with distinct levels of low-temperature (LT) tolerance but same vernalization requirement, and the spring cultivar Chinese Spring.

The transcription profiles of the selected *TaCBF* genes showed that they are induced by cold treatment at 2 °C. Analysis of transcript abundance revealed that the four *TaCBF* genes were expressed at higher levels in the frost tolerant Milena than in the susceptible Russalka. Largest differences (fivefold and fourfold) in expression levels between both winter cultivars were observed in two of the analysed genes, *TaCBF15.2* and *TaCBFA19*, respectively. The higher steady-state expression levels of *TaCBF* genes before the onset of the LT treatment in Milena, combined with stronger induction by cold treatment, suggest that these molecular responses to LT are associated with superior FT development capacity.

The results expand our understanding of the molecular mechanisms underlying LT acclimation in Bulgarian wheat and can be used for development of functional markers for improvement of FT wheat-breeding programmes.

## Introduction

Low temperature (LT) is one of the major abiotic stress factors that limit the growth, productivity and geographical distribution of agricultural crops,[1] including temperate cereals. Even in the established agricultural production areas, seasonal or episodic freezing events can lead to significant losses in yield and quality of wheat. Winter wheat with higher yield and quality potential has to withstand freezing temperatures during the winter. Currently, due to the increased necessity of feeding an ever-expanding human population, one of the main breeding efforts worldwide is the development of wheat cultivars with better adaptation to cold. Greater LT tolerance allows more flexible farm management, environmentally friendly production systems, lower herbicide costs and energy requirements and, finally, increased crop moisture utilization and productivity.[1] Identifying and understanding the genetic and molecular mechanisms of LT adaptation are crucial for improvement of winter wheat survival and thereby the production. Once the LT tolerance pathways and the roles of important cold tolerance genes are elucidated, directed breeding strategies can be developed to increase the LT tolerance in winter wheat.

Autumn-sown winter cereals acquire tolerance to freezing temperatures and become vernalized through exposure to low non-freezing temperatures. The level of accumulated LT tolerance depends on the cold acclimation rate and factors controlling the time of floral transition at the shoot apical meristem.[2] Cold acclimation and vernalization are indispensable for winter wheat cultivars to both survive freezing temperatures in the winter and switch from vegetative to reproductive stage in the spring. During cold acclimation, winter cereals undergo large-scale changes in their transcriptome, which in turn activates the production of an array of proteins to aid the plant's survival during subsequent freezing stress.[3,4]

The vernalization delays flowering until the end of winter and protects the sensitive floral primordia.[5] In wheat this process is regulated by a genetic pathway that involves at least three genes [*TaVRT-1* (*VRN-1*), *VRN-2, TaVRT-2, TaFT1*(*VRN3*)]. The vernalization locus *VRN-1* has been shown to be involved in the LT requirement, and the *Vrn-A1* gene localized on chromosome 5A has been suggested to be a stronger spring/winter habit determinant [6] than the homoelogous *Vrn-B1* (5B) and *Vrn-D1* (5D).[7] Association between the *vrn-1* winter allele and greater freezing tolerance and winter hardiness has been documented.[8,9] Molecular genetic studies have shown very tight genetic linkage between the *VRN1* loci and a major quantitative trait locus (QTL), the frost tolerance 1 (*Fr-1*) locus, associated with resistance to cold stress.[10–12] *Fr-1* has a key role in the freezing tolerance of vegetative tissues. Because the *Fr-1* allele conferring greater freezing tolerance and winter hardiness co-segregates with the *vrn-1* winter allele, it has not been well clarified whether this locus is just a pleiotropic effect of *VRN-*1 or it is a separate gene.[13,14] Later results indicate that the *Vrn–Fr-1* region consists of two distinct, but closely linked loci that control both frost tolerance and vernalization.[15] However, despite the progress in the mapping of the *Vrn1–Fr1* intervals in common wheat, the isolation of the key *Fr-1* gene on this chromosomal region has yet to be done. A second distinct locus, *Fr-2*, affecting freezing tolerance has been detected in a number of wheat and barley mapping populations.[16–19] This locus is located about 30 cM proximal to locus *VRN-*1 on the long arm of chromosome 5.[16–19] *Fr-2* is coincident with a cluster of genes encoding *C-repeat binding factors* (*CBFs*). The *CBFs* are transcriptional activator proteins that regulate pathways affecting cold acclimation and freezing tolerance.[20–23] They belong to the AP2/EREBP transcription factor family and induce *COR* (*cold-regulated*) gene transcription by binding to C-repeat/dehydration-responsive elements (*CRT/DRE*) in the regulatory regions of the *COR* genes.[24] *CBFs* have been implicated in the transcriptional control of many genes, including those involved in phosphoinositide metabolism, biosynthesis of osmolytes, Reactive Oxygen Species (ROS) detoxification, membrane transport, hormone metabolism and signalling.[4,22,25,26]

In addition to their induction by various abiotic stimuli, the expression of *CBFs* is affected by photoperiod [27] and diurnal cycles.[28,29] Some *CBF* genes are expressed at low levels in non-stressed conditions which may lead to continual, albeit low, accumulation of their target genes.[5,15,27,28,30–32] Over-expression of the wheat *CBF* transcription factors increased the freezing tolerance of transgenic plants.[33–35] The relatively large *CBF* families identified in most cereals were divided into four major phylogenetic groups, consisting of two or more sub-groups each.[28] The *CBF* subgroups IIIc, IIId, IVa, IVb, IVc and IVd appear to be unique in *Pooideae*.[28,36,37] Among them, five were suggested to be involved in the development of higher LT tolerance in *Triticeae* crops with a winter growth habit.[28] Several *CBF* genes have been characterized, including 17 from barley,[36] 15 from *T. monococcum*,[37] 37 from hexaploid (6x) wheat (*T. aestivum*) [28] and 11 from rye (*S. cereale*).[29] More recent studies [38] revealed more complex organization of the *CBF* family in 6x wheat, which consists of at least 65 *CBF* genes, 27 of which are paralogs with 1–3 homeologous copies for the A, B and D genomes. Sixty of them were shown to be expressed in the frost tolerant cultivar Norstar. At least 11 different *CBF* genes have been mapped within a small 0.7–0.8 cM region encompassing the *Fr-2* locus in both *T. monococcum* and barley.[18,37,39,40] Subsequently, high-density mapping of this region identified three *CBF* genes (*CBF12*, *CBF14* and *CBF15*) most probably responsible for the observed differences in cold tolerance in *T. monococcum*.[41] Although there are indications that the *CBF* genes cluster on the homeologous group 5 chromosomes of *Triticeae* tribe, and coincide with the QTL for LT tolerance,[16,17,19,29,37,39] it is still not clear which *CBF* genes have a stronger effect on LT tolerance in 6x winter wheat and whether their effect is additive.

In Bulgaria, due to specific agro-climatic conditions, breeding for cold tolerance in 6x wheat has a long tradition. A large number of freezing tolerant cultivars were developed during the last 20 years. However, the genetic mechanism underlying the cold tolerance in Bulgarian wheat is still unknown. Unravelling of the mechanism of natural differences in frost tolerance is an important issue for further improvement of breeding strategies for this trait.

The objective of this study was to identify *TaCBF* genes with major effect on the frost tolerance (FT) of Bulgarian hexaploid winter wheat cultivars. These can prove useful as functional markers in marker-assisted selection for increased frost tolerance.

## Materials and methods

### Plant material

Two wheat (*Triticum aestivum* L.) cultivars, Milena and Russalka, from the gene bank of the Dobrudzha Agriculture Institute (General Toshevo, Bulgaria) as well as Chinese Spring (CS) were used in this study. The winter habit (*vrn-A1/vrn-A1, vrn-B1/vrn-B1, vrn-D1/vrn-D1*) of Bulgarian cultivars and the spring habit of CS (*Vrn-D1/Vrn-D1*) were verified by polymerase chain reaction (PCR) in previous studies [42–44] using the perfect molecular markers developed by Yan et al. [45] and Fu et al.[46] Both Bulgarian cultivars have the same vernalization requirements of 40 days. The photoperiod insensitive phenotypes (*Ppd-D1a*) of both winter cultivars were confirmed by PCR with the allele-specific primers described by Beales et al.[47]

### Frost tolerance test

Screening for frost tolerance of Bulgarian cultivars was performed at the Agricultural Institute, Centre for Agricultural Research, Hungarian Academy of Sciences, Martonvásár (Hungary), following a laboratory method described by Vágújfalvi et al.[19] Seeds were germinated in Petri dishes to achieve the desired size of the seedlings (1.5 cm coleoptile and 1.5 cm roots). They were plotted in wooden boxes in a randomized block design arrangement and were grown in a growth chamber at 15 °C/10 °C (day/night), 75% relative humidity and 260 μmolm^−2^ s^−1^ light intensity. The hardening started when the temperature was reduced to 10 °C/5°C for two weeks, then to 5 °C/0 °C for another two weeks and to +2 °C/−2 °C for one week. Then the plants were gradually subjected to 24 h freezing at two different temperatures, −15 °C and −18 °C. After freezing, the temperature was gradually increased to 17 °C/16 °C and the leaves were cut several centimetres above the soil. Frost tolerance was estimated as the assessment of the re-growth of the plants scored on a scale running from 0 (dead) to 5 (undamaged, tillering plants).

### Plant growth conditions and cDNA synthesis

Seeds of the winter cultivars Milena and Russalka and the spring cultivar CS were germinated on wet filter paper in Petri dishes at 20 °C for 1 day, subsequently at 4 °C for 2 days to synchronize the germination and then moved back at 20 °C for additional 2 days. The seedlings were transferred to hydroponics trays containing modified Hoagland solution [48] for 8 days at 22 °C/16 °C under a 16 h day/8 h night photoperiod, where the illumination stared at 8:00 with a light intensity of 250 μmol m^−2^ s^−1^ and at 73% of humidity in a growth chamber. At the end of this period 1–3 g of the crown tissue (less than 1 cm long sections at the base of the crown) were sampled and individually frozen.

Cold treatment (2 °C) was initiated by changing the temperature in the growth chamber at 8:00 am (with lights on as described above), and maintained for 7 h, followed by transfer to 22 °C for additional 24 h of de-acclimation. Control and cold treated plants were sampled at multiple time points during cold treatment as shown in Table 1. The rapidly harvested crown tissue was immediately frozen in liquid nitrogen and stored at −70 °C until use for RNA extraction.

**Table 1.** Sampling scheme of crown tissue.

Time from initiation of cold treatment	Temperature
0 (control sample) start of the acclimation	22 °С/16 °С
30 min	2 °С
1 h	2 °С
2 h	2 °С
4 h	2 °С
7 h (end of the acclimation)	2 °С
24 h (end of the de-acclimation)	22 °С

RNA was extracted from approximately 100 mg leaf tissue, using Trizol (Invitrogen™) following the manufacturer's instructions. The concentration and quality of RNA were assessed by NanoDrop 2000 (Thermo Scientific) and by gel electrophoresis, respectively. The RNA was treated with TURBO DNase (Ambion®) to remove possible DNA contamination in RNA samples. The absence of genomic DNA was confirmed by gel electrophoresis and control real-time quantitative reverse-transcription PCR (qRT-PCR) reactions lacking reverse transcriptase enzyme. First strand cDNA synthesis was performed on 5 μg total RNA, using RevertAid H Minus M-MuLV reverse transcriptase (Fermentas, 50 U), Ribonuclease Inhibitor (Fermentas, 40 U), oligo (dT)18 primers (1 μg) in 20 μL. First strand cDNA samples were diluted in water to a concentration of 40 ng/μL and stored at −70 °C until use.

### Real-time qRT-PCR

The transcript accumulation of each gene was detected by qRT-PCR using TaqMan fluorescence detection on a 7300 Real-Time PCR System (Applied Biosystems®). The gene-specific primers and the fluorescent TaqMan probes were designed by Primer Express® Software Version 2.0 (Applied Biosystems®) using the nucleotide sequences of the selected *CBF* genes (EF028764, EF028766, EF028769 and EF028775) [28] (Table 2). Both gene-specific primers and the dual labelled TaqMan probes (FAM and TAMRA) were purchased from Applied Biosystems®.

**Table 2.** Primers used in qRT-PCR.

Genes	Primers	Sequence	Amplicon (bp)
*TaCBFIIId-15.2*	F	5′-GATGACGGAAGCTGTAGCCAAT-3′	102
	R	5′-AAACATTCCGTCGCCAGAAG-3′	
	Probe	5′-TACCGGCTCGTCGTG-3′	
*TaCBFIIId-A19*	F	5′-GCCTCTGGAGCTACTGATGTCTG-3′	68
	R	5′-CAATCGGGATAGCAAAAATCCTC-3′	
	Probe	5′-ACCTCCAGTGGGTTC-3′	
*TaCBFIVa-A2*	F	5′-GCCGGAAGCCGGAGTTA-3′	61
	R	5′-CCGCCATCTGGGCACT-3′	
	Probe	5′-TCTGGCTTGGCACCT-3′	
*TaCBFIVb-D21*	F	5′-TCATCGGAGCCCCAATCA-3′	56
	R	5′-CGTGCGTTAAGCCGTTGTG-3′	
	Probe	5′-TCGAACCTCGCCAGTC-3′	
*wact2*	F	5′-CGTGTTGGATTCTGGTGATG-3′	87
	R	5′-AAGGTCAAGACGAAGGATGG-3′	
	Probe	5′-CTGTGCCAATCTATGAAGGATATGCCC-3′	

PCR mix was performed in a total volume of 25 μL containing 120 ng single-stranded cDNA as a template, 12.5 μL 2xTaqMan Universal PCR Master mix (Applied Biosystems®), 0.9 pmol/μL of each forward and reverse primer and TaqMan probe (0.2 pmol/μL). The cycling conditions were as follows: 10 min at 95 °C, followed by 50 cycles of 95 °C for 15 s/60 °C for 60 s. All PCR reactions were repeated twice.

The constitutively expressed gene α-actin (CJ902800) [49] was used as an internal control. Raw *Ct* values (difference in the number of PCR cycles required to reach the log phase of amplification) from two repetitions were imported into BioRad Gene Expression Macro Version 1.1 software for expression analysis. The relative expression level was determined by using the 2^−ΔΔCt^ method,[50] where the *Ct* values of the target gene, normalized to actin (Δ*Ct*), were expressed relative to the *Ct* values of the ‘calibrator’ CS at 0 h.

## Results and discussion

### Frost tolerance rank of Bulgarian winter wheat cultivars

**Figure 1.**
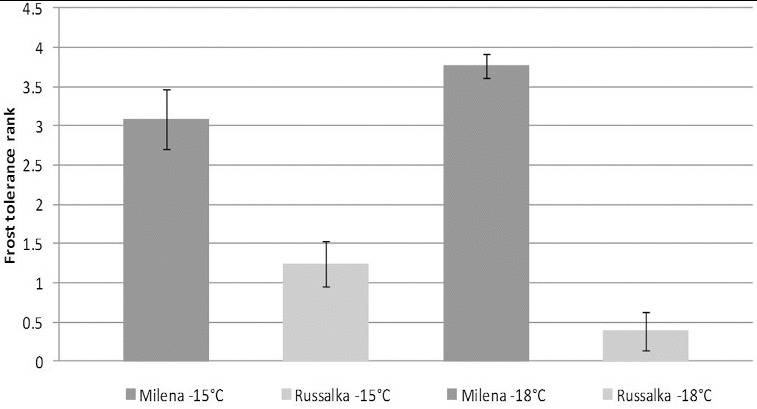
Frost tolerance rank (score 0–5) at −15 °С and −18 °С of two studied commercial Bulgarian wheat cultivars. Student's *t-*test was used to test for statistical significance (*p* < 0.05).

The frost tolerance test of the selected cultivars with winter growth habit (*vrn-1, vrn-3*) was performed in Hungary (2008) under controlled conditions, using two freezing temperatures (−15 ºC and −18 ºC). According to the results from the frost test, Milena proved to be highly frost tolerant, while Russalka was identified as frost sensitive (Figure 1). These results were confirmed by Tsenov et al. [51] and Ganeva et al. [52] through a multi-year frost tolerance test using the field-laboratory method [53] at the Dobrudzha Agricultural Institute, G. Toshevo, Bulgaria. Both authors showed that cv. Milena is highly tolerant to all freezing temperatures tested (−18 ºC and −21 °C) after both sufficient and insufficient cold acclimation temperatures. Cluster analysis of cold tolerance test data [51] placed Milena in the clade of the highly tolerant Russian cv. Mironovskaya 808, while Russalka was clustered near to the sensitive Italian spring cultivar San Pastore.

### Expression of *CBF* genes in wheat cultivars with different frost tolerance capacity

High-density mapping strategies, microarray analysis and quantitative RT-PCR have been extensively used to identify possible candidate genes for frost tolerance in both hexaploid and diploid wheat.[16,19,30,54] Among different signalling pathways, the *CBF* regulon plays a pivotal role in cold acclimation of wheat and other cereal crops. The fine mapping of the *Fr-2* locus on chromosome group 5 in wheat revealed a complex structure including multiple *CBF* genes.[16,19,30,37] According to Badawi et al.,[28] the cluster of *CBF* genes at *Fr-2* loci in hexaploid wheat includes *CBF*-2, -3, -4, -6, -9, -10, -12, -14, -15, -19, -20, -21 and -22. Knox et al. [41] described that *T. monococcum CBF* genes at the *Fr*-2A locus are organized in three subclusters, each composed of 3 or 4 genes: proximal (*CBF*-2, -4, -9 and -17); central (*CBF* -14, -15, -12) and distal (*CBF* -16, -13, -3 and -10). These authors suggested that the locus for frost tolerance is linked to the central gene subcluster (*CBF*-14, -15, -12) and *CBF*-6, -19, -20, -21 and -22 do not belong to the frost tolerance gene cluster. Studying the transcription profiles of different *T. aestuvum CBF* genes using a quantitative PCR system, Vágújfalvi et al. [30] have found that most of the genes at this locus are induced by cold treatment at 2 °C for 2 h, with *TaCBF-14*, -*15* and -*16* having the highest transcript levels. These data have been confirmed later by Båga et al. [16], who reported that during cold acclimation *TaCBF-14* and -*15* are expressed at higher level in the cold tolerant Norstar than in the sensitive winter Manitou.

In this study two winter cultivars (Milena and Russalka) selected for their distinct FT (Figure 1) and the spring cultivar CS were chosen to study the expression pattern of four *TaCBF* genes during cold acclimation at 2 °C, using qRT-PCR analysis. The four *TaCBF* genes (*CBF15-5AL*, *CBFA19-5AL CBFA2-5AL* and *CBFD21.1-5DL*), belonging to the groups *CBFIIId*, *CBFIVa* and *CBFIVb*, were chosen on the basis of published information, suggesting their importance in the development of FT in wheat.[28]

**Figure 2.**
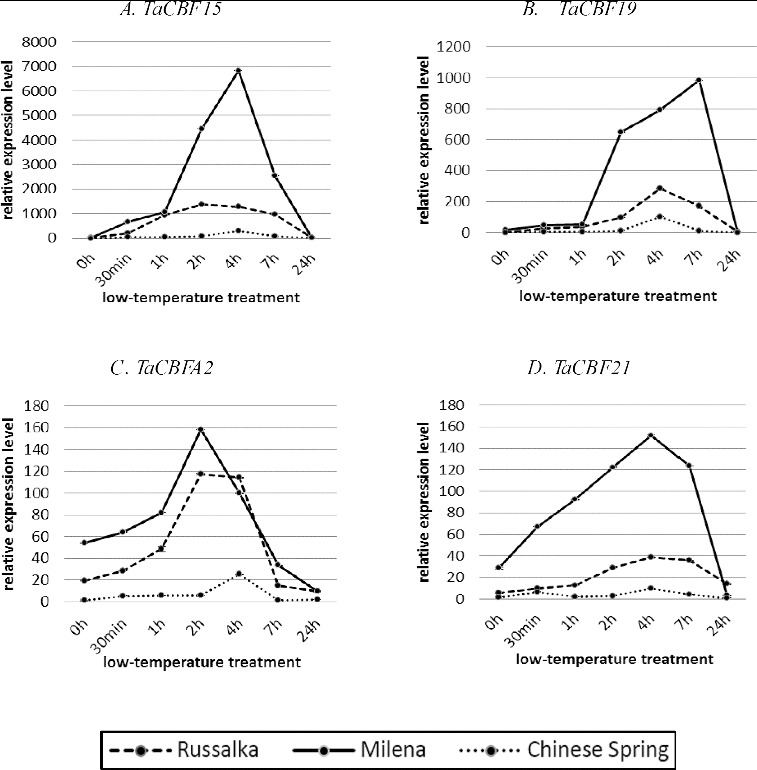
Quantitative RT-PCR expression analysis of *TaCBF* genes in highly tolerant winter cultivar Milena, susceptible winter cultivar Russalka and the frost sensitive spring cultivar CS. *TaCBF15* (**A**); *TaCBF19* (**B**); *TaCBFA2* (**C**); *TaCBF21* (**D**). Time points shown are 0 h (control condition at 22 °C); 30 min, 1 h, 2 h, 4 h, 7 h (LT stress at 2 °C); and 24 h (de-acclimation).

The expression results from qRT-PCR (Figure 2) showed that *TaCBF* genes are induced by LT and, with the exception of *CBFA19*, attained maximum level of expression between the second and fourth hours of acclimation at 2 °C. Following the kinetics of the transcription, the *CBF* genes studied here demonstrated different patterns of relative transcript abundance between the highly tolerant and the susceptible winter cultivar as well as between the two winter cultivars and CS.

The qRT-PCR analysis revealed that during cold acclimation the expression level of the group IIId genes (*CBF15-5AL*, *CBFA19-5AL*) was more pronounced than that of the group IVa (*CBFA2-5AL)* and IVb ones (*CBFD21.1-5DL*), although higher transcript accumulation of all studied genes was associated with the higher capacity of the winter cultivars to develop LT tolerance (Figure 2).

Among the *CBF* genes analysed in this study, the highest level of accumulation was observed for *CBF15-5AL* with maximum transcript abundance at 4 h of LT treatment in the tolerant winter cv. Milena and earlier, at 2 h, in the susceptible winter cv. Russalka. It is worth noting that the transcript accumulation in Russalka reached a plateau between 2 and 4 h and the decline thereafter was slower than in the other two analysed cultivars. Induction of *CBF15-5AL* expression with a peak at 4 h was also observed in CS. However, the pattern was different and the transcript abundance in this cultivar was up to 20- and 50-fold lower than that in Russalka and Milena, respectively (Figure 2(A)). Similar results for the tolerant winter-hardy cultivar Mir808 have been obtained by Motomura et al.,[32] who reported a transient increase in *TaCBFA15* expression with a maximum between the fourth and sixth hours of the LT, resembling the pattern obtained for Milena in the present study. On the other hand, our results for CS disagree with those of Motomura et al.,[32] who found no increase in the transcript abundance of *TaCBF15* in the leaf tissue of this cultivar during LT course treatment at 4 °C. The differences in the expression patterns observed in both studies might be a result of the different acclimation temperatures and/or tissues used. The peak expression level in the tolerant Milena was 5-fold higher in comparison to the highest expression level in the susceptible winter Russalka and 22-fold higher compared to CS, while the difference between the susceptible winter Russalka and CS was only 4.5-fold. Vágújfalvi et al. [30] identified that under cold acclimation at 2 °C *CBF* genes from the central cluster of the *Fr2* locus (*TaCBF14*, -*15* and -*16*) were characterized with the highest transcript levels and those levels were more than fourfold higher in lines carrying the frost tolerant *Fr-A2* allele than in those with a frost sensitive one. Up-regulation of the transcript levels of *TmCBF16*, *TmCBF12* and *TmCBF15* at mild cold temperatures (15 °C) has been reported by Knox et al. [41] only in the frost tolerant wheat G3116 but not in the frost sensitive Dv92. In contrast, no expression of *TaCBF15* has been identified in the crown and leaf tissues of winter tolerant (Harnsek and Solstice) and spring (Paragon) cultivars under both prolonged acclimation and shock experiments in a microarray analysis,[4] which implies that in different wheat genetic backgrounds distinct *CBF* genes or *CBF* expression levels are responsible for the development of different levels of freezing tolerance.

High level of expression was also observed for *TaCBFA19*, although the extent of accumulation of its transcripts was lower than that of *TaCBFA15* (Figure 2(B)). The expression of *TaCBFA19* was strongly induced in the tolerant cultivar Milena at the first hour and continued to increase up to seven hours. In contrast, the susceptible winter cultivar Russalka showed a much slower increase in transcript abundance after the first hour with a peak at the 4th hour and a decrease thereafter. Expression in the spring cultivar CS followed the pattern of Russalka. The peak level in the tolerant Milena was 3.5-fold higher in comparison to the highest expression level in the susceptible winter cv. Russalka. Both winter cultivars showed higher maximum expression level, 9.45-fold and 2.7-fold, respectively, as compared to CS. Our results are in accordance to those of Sutton et al.[54] who found that *TaCBF19* is differentially expressed between frost tolerant and frost susceptible mutant winter lines of wheat. Both results confirm the involvement of this gene in the development of superior frost tolerance. However, they are in contrast to the study of Knox et al.,[41] who identified this gene as not belonging to the frost tolerance gene cluster.

In comparison to *TaCBFA15* and *-A19*, the expression level of the other two genes, *TaCBFA2* and *TaCBFD21*, was much lower. However, the induction of *TaCBFA2* was faster in both winter cultivars and peaked at the second hour of the cold acclimation (Figure 2(C)), while the pattern of its accumulation in CS was distinct with a peak at the 4 h of the cold acclimation. While in the sensitive winter cv. Russalka the transcript abundance remained at the same level up to 4 h of cold acclimation, in the highly tolerant cultivar Milena the expression of *TaCBFA2* dropped quickly after the peak at 2 h. On the basis of microarray analysis, Sutton et al. [54] suggested the involvement of *TaCBFA2* in the control of expression of a cluster of *Cor/Lea* genes on 5A. Therefore, the observed differences in the expression patterns in the two winter cultivars analysed in the present study might be related to distinct regulation of downstream genes, leading to differences in their frost tolerance capacity. However, more detailed studies are needed to further test this hypothesis. The maximum expression level of *TaCBFA2* in the frost tolerant Milena was 1.35-fold higher than that in the susceptible Russalka and 27-fold higher than that in the spring cv. CS.

The role of *TaCBFA2* in LT tolerance was first proposed by Badawi et al.,[28] who, using spring and winter isogenic wheat lines, demonstrated its down-regulation by the presence of dominant *Vrn-1* alleles. Later studies [29,41,54] confirmed the involvement of this gene in the cold acclimation and frost tolerance development in wheat and rye. Significantly higher *CBF2* and *CBF4* transcript levels have been also observed in the more freezing tolerant winter barley cultivar Nure in comparison to the less tolerant spring cultivar Tremois.[27] Differential expression of *TaCBFA2* in both crown and leaf tissues between the tolerant winter and spring genotypes under prolonged and LT shock experiments has been also documented in both microarray analyses performed by the group of Winfield et al.[4]

In our study, the qRT-PCR analysis of *TaCBFD21* showed maximum transcript accumulation 4 h after the beginning of CA, which declined thereafter in all analysed cultivars. However, its relative transcript accumulation was much faster in the highly tolerant winter cultivar in comparison to both Russalka and CS (Figure 2(D)). Its expression at 4 h of LT treatment in Milena was about 4-fold higher than in Russalka and 15-fold higher than in CS.

The genetic capacity of winter cultivars to induce higher levels of expression during LT response was also reflected in the constitutive levels measured under control growth conditions at warmer temperatures (22 °C). Since LT is not involved in the regulation of the expression, the higher levels in winter cultivars versus spring is a sign of the different capacities to express *CBF* genes and is a consequence of the difference in the capacity of winter cultivars to develop FT. Badawi et al. [28] reported a high and more sustained expression for most *CBFIIId*, *CBFIVa*, *CBFIVb*, *CBFIVc* and *CBFIVd* genes. In addition, these groups showed higher basal expression in the winter cultivar versus the spring one. In *Arabidopsis*, the transgenic constitutive over-expression of the *CBF* genes results in an increase in freezing tolerance even without prior cold acclimation,[55–57] although this also results in a slow-growing dwarf phenotype, as a consequence of interactions between *CBF* genes and the gibberellins biosynthetic pathway.[58]

In our study, the *CBF* genes from groups IIId and IVb showed between 5- and 10-fold higher steady-state expression level at 22 °C in the highly frost tolerant cultivar Milena (Figure 2(A)–(C)) as compared to cv. Russalka and, respectively, more than 12-fold higher steady-state expression level in comparison to the frost sensitive spring cultivar CS. Noticeable steady-state *CBFA2* transcript levels (group IVa) were observed in the studied winter cultivars (19-fold and more) in comparison to CS. However, the difference in the induction levels of *CBFA2* in winter cultivars was not as obvious as that between them and CS. Sharp differences in mRNAs steady-state levels between the frost tolerant winter cv. Cheyenne and the frost sensitive spring cultivar CS have been reported.[30] This suggests that the fast and/or the higher cold *CBF* induction might be associated with the differences in the frost tolerance capacity between cultivars. Several reports [15,27–29,31] have also described that some *CBF* genes are expressed at low levels in non-stressed conditions in barley, wheat and rye and may lead to continual, albeit low accumulation of the target genes.

Our results suggest that the higher *TaCBF- A15*, *-A19*, *-A2* and *-D21.1* expression is potentially associated with the winter cultivar's superior FT development capacity. They also imply that in the different genetic backgrounds, different genes along the *CBF* cluster might be implicated in determination of cultivar differences in FT. Additional research aimed at achieving more detailed understanding of the genetic cascades and the complex interaction of genes involved in the development of superior LT tolerance in wheat is highly necessitated.

## Conclusions

The comparative quantitative expression analysis (qRT-PCR) showed that the four selected *CBF* genes are induced and expressed at higher levels in the highly frost tolerant Bulgarian winter cultivar Milena as compared to the more susceptible winter cultivar Russalka. The observed differences in the level of transcription activity of the *CBFA15.2*, *CBFA19*, *CBFD21* and *CBFA2* genes closely correspond to the frost tolerance (LT_50_) of both winter cultivars. The selected genes could be used as potential functional markers for marker-assisted selection of cold tolerant wheat cultivars carrying *Fr-2* alleles.

Functional characterization of other *CBF* genes and alleles and identification of the *CBF* genes responsible for better FT capacity of wheat as well as the generation of improved haplotypes combining favourable *CBF* alleles may provide a useful tool to enhance frost tolerance and hardiness in Bulgarian wheat-breeding programmes. 
